# Antifatigue Effects of* Antrodia cinnamomea* Cultured Mycelium via Modulation of Oxidative Stress Signaling in a Mouse Model

**DOI:** 10.1155/2017/9374026

**Published:** 2017-03-23

**Authors:** Yange Liu, Lanzhou Li, Shengshu An, Yuanzhu Zhang, Shiwei Feng, Lu Zhao, Lirong Teng, Di Wang

**Affiliations:** School of Life Sciences, Jilin University, Changchun 130012, China

## Abstract

*Antrodia cinnamomea*, a folk medicinal mushroom, has numerous biological effects. In this study, we aim to assess whether the antifatigue effects of* A. cinnamomea *mycelia (AC) and its underlying mechanisms are related to oxidative stress signaling using behavioral mouse models and biochemical indices detection. Mice were orally treated with AC at doses of 0.1, 0.3, and 0.9 g/kg for three weeks. AC had no effect on the spontaneous activities of mice indicating its safety on central nervous system. Furthermore, results obtained from weight-loaded forced swimming test, rotary rod test, and exhausted running test confirmed that AC significantly enhanced exercise tolerance of mice. Biochemical indices levels showed that these effects were closely correlated with inhibiting the depletion of glycogen and adenosine triphosphate stores, regulating oxidative stress-related parameters (superoxide dismutase, glutathione peroxidase, reactive oxygen species, and malondialdehyde) in serum, skeletal muscle, and liver of mice. Moreover, the effects of AC may be related with its regulation on the activations of AMP-activated protein kinase, protein kinase B, and mammalian target of rapamycin in liver and skeletal muscle of mice. Altogether, our data suggest that the antifatigue properties of AC may be one such modulation mechanism via oxidative stress-related signaling in mice.

## 1. Introduction

Fatigue, a complex symptom, is accompanied by difficulty in initiating or sustaining activities, resulting from severe stress, mental work, and/or hard physical labour [1]. Liver serves as a reservoir for blood, glycogen, fats, carbohydrates, and even proteins, where protein synthesis and substance metabolism are performed. Nutrients from liver are released into the blood and further to muscle, contributing to maintain muscle function under movement conditions and delay the occurrence of fatigue [2, 3].

There are four theories (“exhaustion theory,” “radical theory,” “protective inhibition theory,” “clogging theory,” and “hemoglobin theory”) to explain the pathomechanisms of fatigue, among which “radical theory” has been widely accepted recently [4]. Due to the high intensity or exhaustive exercise, free radicals are reportedly formed in limb muscle and in vitro contraction [5]. Overproduction of a large amount reactive oxygen species (ROS), in turn, leads to oxidative stress by causing imbalance between oxidant and antioxidant defense system (glutathione peroxidase, glutathione peroxidase, and superoxide dismutase), which carry the body in a risk of injury via affecting the homeostatic environment [6]. Importantly, ROS leads to fatigue by damaging muscles, especially in the diaphragm [5]. Evidence provided further supports that not only depressing depletion of energy source had positive effects on antifatigue, but also inhibiting free radical generation enhanced exercise performance [7]. Adenosine 5′-monophosphate- (AMP-) activated protein kinase (AMPK) is very sensitive to the ratio of AMP/ATP, leading to regulating the absorption of glucose and fatty acids [8]. Furthermore, high glucose activates mammalian target of rapamycin (mTOR) signaling, therefore, enhances the anabolic response of protein and lipid [9].

Current studies focus on searching health agents from natural products for postponing fatigue, improving athletic ability, and accelerating the elimination of fatigue-related metabolites abilities [10]. As reported, fungus and herbs have been widely and effectively used to enhance fatigue endurance. Green tea polysaccharides significantly inhibit the oxidative stress induced by the exhaustive exercise and alleviated exercise-induced fatigue [11].* Antrodia cinnamomea*, a folk medicinal mushroom, is a native rare and precious species in Taiwan only [12]. Due to its various pharmacological activities,* A. cinnamomea *has been accepted worldwide, which accelerates its insufficiency on resource [13].* A. cinnamomea *mycelium obtained by submerged fermentation can serve as an excellent alternation to fruit bodies and further expand its application. As reported previously,* A. cinnamomea *possessed antioxidant [12], anti-inflammatory [14], anti-tumor [15], and hepatoprotection [16] activities.* A. cinnamomea *fruit body elevated swimming endurance in mice via increasing blood glucose concentration and hepatic and muscular glycogen deposition [10]. Through modulation oxidant system, especially the activities of SOD and GSH-Px,* A. cinnamomea *successfully applied to treat free radical-related inflammation [17]. However, the potential antifatigue activities of* A. cinnamomea *mycelium and its underlying mechanisms have not been systematically reported yet.

The present study aims to evaluate the potential antifatigue effects of* A. cinnamomea *mycelium in mice. During the whole experiment, the concentration of ATP and the activities of oxidant related enzymes in serum, skeletal muscle, and liver were detected. To analyze its underlying mechanism, relevant signaling activations including protein kinase B (Akt), AMPK, and mTOR in liver and skeletal muscle were measured.

## 2. Materials and Methods

### 2.1. *A. cinnamomea* Submerged Fermentation

*A. cinnamomea* (ATCC 200183) was obtained from the American Type Culture Collection (Virginia, USA) and cultured in a defined liquid medium as follows: 20 g/L of glucose, 10 g/L of tryptone, 10 g/L of yeast extract powder, 1.25 g/L of KH_2_PO_4_, 0.65 g/L of MgSO_4_, and 0.12 g/L of vitamin B_1_ with an natural pH, and it was incubated at 25°C for 7 days.

*A. cinnamomea* mycelia (AC) was obtained after centrifugation and lyophilization for further experiments. As measured previously, AC contains 11.7% of total sugar, 30.6% of total protein, 30.1% of crude fat, 8.1% of triterpenoids, 5.4% of mannitol, 0.35% of flavonoids, and 0.16% of adenosine, which were measured using 3,5-dinitrosalicylic acid colorimetric assay [18], Kjeldahl method [19], petroleum benzene extraction method, vanillin-glacial acetic acid and perchloric acid colorimetric spectrophotometry [20], periodate oxidation method [21], erinitrit-aluminium trichloride assay [18], and high-performance liquid chromatography analysis [18], respectively.

### 2.2. Animals Experiments Process

**Figure 1:**
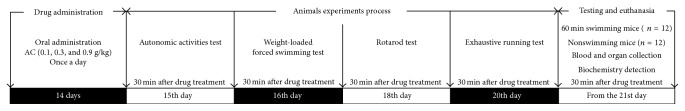
The experimental protocol and drug administration.

The experiments were approved by the Institution Animal Ethics Committee of Jilin University (Reference number 2015-003). 96 Kunming (KM) mice (8 weeks; 18–22 g; half male and female) were maintained under standard conditions (temperature of 22°C ± 2°C, humidity of 50%, and 12 h light-dark cycle). Mice were randomly divided into 4 groups (*n* = 24/group;* F *= 0.014–1.37; half male and female) and orally treated with 10 mL/kg of normal saline (CTRL) and AC at doses of 0.1, 0.3, and 0.9 g/kg daily for three weeks. The detailed experimental protocol and drug administration are shown in Figure 1.

#### 2.2.1. Autonomic Activities Test

Independence activity test was performed according to the method described previously with some modification [22]. Thirty minutes after AC administration, mice were placed individually in the autonomic activities instrument (ZZ-6, Chengdu Taimeng Science Technology Co., Ltd., Chengdu, China). Locomotor activities and enabled vertical movements, including jumping, horizontal movements, and walking and running, were counted.

#### 2.2.2. Weight-Loaded Forced Swimming Test (FST)

Thirty minutes after AC administration, test was carried out with mice loaded with a lead block (10% of bodyweight) attached to their tails in water maintained at 25 ± 2°C. Mice loss coordinated movements and failure to swim to the surface within 10 sec were used to measure their exhaustion. The time of exhaustive swimming was recorded.

#### 2.2.3. Rota-Rod Test (RRT)

Thirty minutes after AC administration, the rotarod test was conducted as previous research with minor modification [23]. Before the formal test, mice were trained twice on rotarod at 15 rpm for 60 s to adapt to the instrument (ZB-200, Chengdu Taimeng Science Technology Co., Ltd., Chengdu, China). And, then, mice were placed inside a rotarod spinning and allowed to run at speed of 15 rpm until they were exhausted and dropped from the rod. The total running time was recorded.

#### 2.2.4. Exhaustive Running Test (ERT)

Following previous description [24], thirty minutes after AC administration, mice were trained twice on the runway at 20 rpm for 5 min to adapt to the treadmill (FT-200, Chengdu Taimeng Science Technology Co., Ltd., Chengdu, China). Exhaustion was determined by failing to return to the runway within 15 sec and losing dynamism of movements. The exhaustive running time was recorded.

### 2.3. Samples Collection and Biochemical Indexes Measurement

Thirty minutes after the final AC administration, half of mice (*n* = 12/group; half male and female) were separately placed in the swimming pond (diameter 20 cm, depth 50 cm, and temperature 25 ± 2°C) and swam for 60 min without loads, carried out as described in previous reported with minor modification, developing a swim-training model [25]. Another half of mice (*n* = 12/group; half male and female) did not receive any treatment during 60 min. Blood, liver and skeletal muscle were collected and immediately placed in −80°C.

Levels of alanine aminotransferase (ALT) and aspartate aminotransferase (AST) in the serum, levels of glycogen and ROS in liver and skeletal muscle, and levels of adenosine triphosphate (ATP), GSH-Px, SOD, and malondialdehyde (MDA) in serum, liver, and skeletal muscle were determined according to the protocol recommended by the commercial diagnostic kits purchased from Nanjing Jiancheng Institute of Biotechnology Co. Ltd. (Nanjing, China).

### 2.4. Western Blot

Liver and skeletal muscle tissues were homogenized, lysed, and measured total protein concentration by BCA protein assay kit (Merck Millipore, Germany). Samples containing 40 *μ*g of total protein were loaded and separated by 12% SDS–PAGE (Bio-Rad, USA), transferred to nitrocellulose membrane (0.45 *μ*m; Millipore, USA), and blocked for more than 4 h with 5% bovine serum albumin (BSA)/TBS buffer. Immunoblotting was detected using primary antibodies including phosphor- (P-) Akt (07-1398), total- (T-) mTOR (04-385), P-mTOR (09-213) (Merck Millipore, Darmstadt, Germany), T-Akt (ab131443), T-AMPK (ab133348), and P-AMPK (ab133348) (Abcam, Cambridge, USA) and glyceraldehyde-3-phosphate dehydrogenase (GAPDH) (sc-25778) (Santa Cruz Biotechnology, Santa Cruz, USA) via incubation overnight at 4°C followed by washing in TBST buffer containing 5% BSA and 0.1% Tween-20. The primary antibody was detected with a HRP-conjugated goat anti-rabbit secondary antibody (sc-3836) (Santa Cruz Biotechnology, Santa Cruz, USA) and visualized by Gel Imaging System (UVP, California, USA). The bands density was quantified using Image J (National Institutes of Health, Bethesda, USA) via calculating the average optical density.

### 2.5. Statistical Analysis

Data were expressed as mean ± SEM. A one-way analysis of variance (ANOVA) was used to detect statistical significance followed by post hoc multiple comparisons (Dunn's test) using SPSS 16.0 software (IBM corporation, Armonk, USA).* P* values less than 0.05 were considered significant.

## 3. Results

### 3.1. Effects of AC on Mouse Exercise Capacity

**Figure 2:**
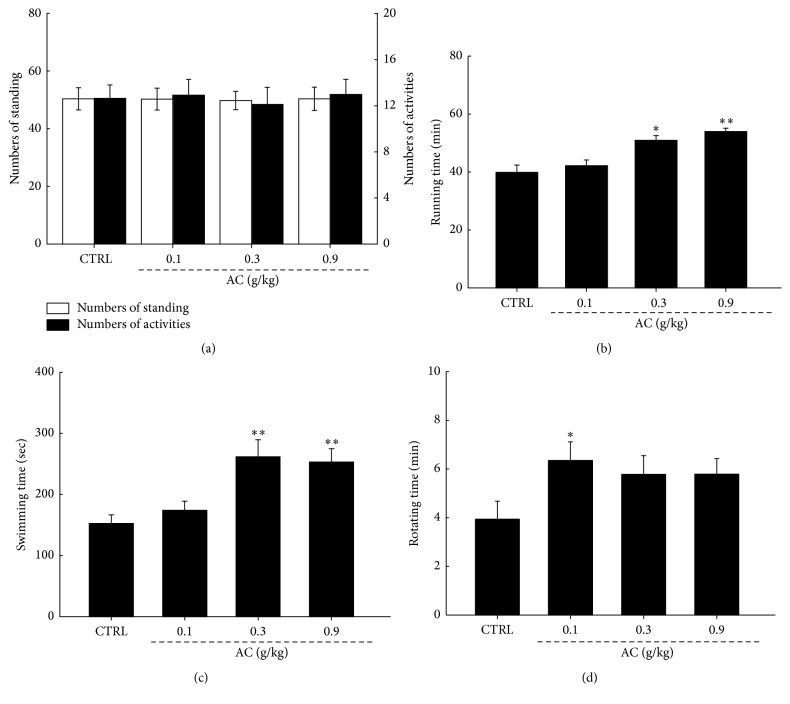
Three-week AC treatment brought no significant differences in movements (a) but significantly prolonged exhaustive time in (b) running, (c) swimming, and (d) rotating tests. The data were expressed as means ± SEM (*n* = 24) and analyzed using a one-way ANOVA. ^*∗*^*P* < 0.05 and ^*∗∗*^*P* < 0.01 in a comparison with the control mice.

Behaviors were measured to investigate the effect of AC on mouse exercise capacities. Compared to control mice, three-week AC treatment brought no significant differences in numbers of standing and activities indicating its safety on mice (*P* > 0.05,* F* = 0.03–1.47; Figure 2(a)). FST, RRT, and ERT are classic rodent models to evaluate the capacity of antifatigue [24]. AC at doses of 0.3 and 0.9 g/kg strongly enhanced exercise capacity of mice in ERT and FST (*P* < 0.05, Figures 2(b) and 2(c)). 0.9 g/kg of AC prolonged over 35% of exhaustive time compared to that of control mice in ERT (53.9 min versus 39.8 min; *P* < 0.01,* F* = 10.23; Figure 2(b)). In FST, 0.3 and 0.9 g/kg of AC resulted in over 66% enhancement on swimming time compared to that of control mice (261.5 s and 252.8 s versus 152.4 s; *P* < 0.01;* F* = 10.85 and 11.15; Figure 2(c)). The retention time in rotating exercise was significantly increased by AC at 0.1 g/kg (6.4 min versus 3.9 min; *P* < 0.05;* F* = 5.09; Figure 2(d)). Detailed data can be found in Table  1s in Supplementary Material available online at https://doi.org/10.1155/2017/9374026.

### 3.2. Effects of AC on Liver Function

**Figure 3:**
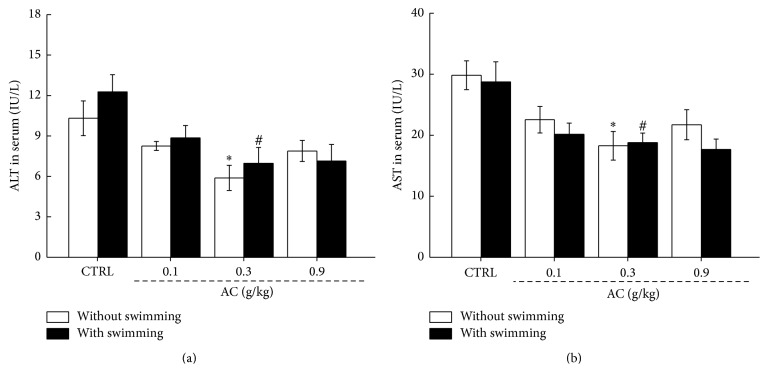
Three-week AC treatment reduced the activities of sera (a) ALT and (b) AST of mice with or without 60 min swimming. The data are expressed as means ± SEM (*n* = 12) and analyzed using a one-way ANOVA. ^*∗*^*P* < 0.05 in a comparison with the control mice without swimming; ^#^*P* < 0.05 in a comparison with the control mice with 60 min swimming.

The levels of ALT and AST in the serum are considered as a biochemical marker for assessing liver function [26], which were examined to explore effect of AC on hepatic function. Compared to unexercised mice, 60 min swimming failed to influence the levels of ALT and AST in serum (*P* > 0.05,* F* = 0.18–1; Figure 3). In mice without swimming, AC at 0.3 g/kg strongly reduced the levels of ALT (5.89 IU/L versus 10.31 IU/L; *P* < 0.05,* F* = 5.63; Figure 3(a)) and AST (18.27 IU/L versus 29.83 IU/L; *P* < 0.05,* F* = 4.43; Figure 3(b)). In mice with 60 min swimming, AC at 0.3 g/kg showed similar reductive effects on the serum levels of ALT (6.97 IU/L versus 12.27 IU/L; *P* < 0.05,* F* = 6.5; Figure 3(a)) and AST (18.77 IU/L versus 28.74 IU/L; *P* < 0.05,* F* = 5.86; Figure 3(b)). Detailed data can be found in Table  2s.

### 3.3. Effects of AC on Levels of Glycogen and ATP

**Figure 4:**
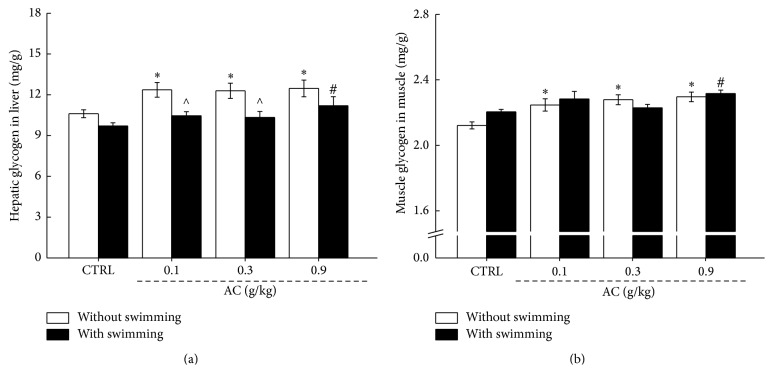
Three-week AC treatment enhanced the levels of (a) hepatic glycogen and (b) skeletal muscle glycogen of mice with or without 60 min swimming. The data are expressed as means ± SEM (*n* = 12) and analyzed using a one-way ANOVA. ^*∗*^*P* < 0.05 in a comparison with the control mice without swimming; ^#^*P* < 0.05 in a comparison with the control mice with 60 min swimming; ^∧^*P* < 0.05 in a comparison between the same agent treated mice with and without 60 min swimming.

Depletion of glycogen is the primary factor in fatigue and exhaustion during exercise [27, 28]. Compared to unexercised mice, low hepatic glycogen levels were observed in mice after 60 min swimming (*P* < 0.05,* F* = 4.85 and 5.3; Figure 4(a)); however, no significant changes on muscle glycogen were noted in exercised mice (*P* > 0.05,* F* = 0.02–0.36; Figure 4(b)). AC strongly enhanced the levels of hepatic glycogen and muscle glycogen in mice with and without swimming (*P* < 0.05,* F* = 4.39–6.11; Figure 4), but failed to influence the changes caused by exercise stimuli. In mice with 60 min swimming, AC at 0.9 g/kg enhanced 15.5% of hepatic glycogen (11.2 mg/g versus 9.7 mg/g; *P* < 0.05,* F* = 5.8; Figure 4(a)) and 5.4% of muscle glycogen (2.33 mg/g versus 2.21 mg/g; *P* < 0.05,* F* = 4.39; Figure 4(b)). Detailed data can be found in Table  3s.

ATP promotes transmission with adenosine diphosphate (ADP) to supply energy for the vital activities [29]. Interestingly, compared to nonswimming mice, ATP levels in serum, liver, and skeletal muscle were enhanced after 60 min swimming (*P* < 0.05,* F* = 5.04–12.3; Table 1). Although AC increased ATP levels compared to nontreated mice, it failed to regulate the tendency caused by exercise stimuli. In mice without swimming, AC at dose of 0.9 g/kg resulted in over 20% increment of ATP levels in serum, liver, and skeletal muscle (*P* < 0.05,* F* = 4.61–15.07; Table 1). Differently, in mice with swimming, AC only significantly enhanced the ATP levels in serum and skeletal muscle (*P* < 0.05,* F* = 5.7–11.36; Table 1). AC at 0.9 g/kg resulted in 58% enhancement in serum (*P* < 0.01,* F* = 10.88; Table 1) and 26.8% enhancement in skeletal muscle (*P* < 0.01,* F* = 11.36; Table 1).

### 3.4. Effects of AC on the Levels of Oxidation Factors

Intense exercise is accompanied by the imbalance on oxidation system [30, 31]. Compared to nonswimming mice, the accumulation of MDA in serum and overproduction of ROS in liver and skeletal muscle were observed in mice with 60 min swimming (*P* < 0.05,* F* = 5.3–12.58; Table 2), and AC failed to reverse these changes caused by exercise stimuli. In skeletal muscle, 60 min swimming caused a reduction on the levels of GSH-Px compared to nonswimming group (*P* < 0.05,* F* = 4.8–6.22; Table 2).

In mice without exercise, AC strongly suppressed the ROS accumulation in liver and skeletal muscle (*P* < 0.05,* F *= 4.75–11.31; Table 2), reduced MDA levels (*P* < 0.05,* F *= 4.97–6.6; Table 2) in serum, liver, and skeletal muscle, and enhanced the SOD activates (*P* < 0.05,* F *= 4.82–30.49; Table 2) in serum, liver, and skeletal muscle. AC enhanced over 15% GSH-Px levels in serum and skeletal muscle (*P* < 0.05,* F *= 5.56–14.27; Table 2), but not in liver.

In mice with 60 min swimming, over 12% reduction on MDA levels in serum and tissues (*P* < 0.05,* F* = 4.2–4.99; Table 2) and over 13% inhibition on ROS levels in liver and skeletal muscle (*P* < 0.05,* F* = 4.59–5.72; Table 2) were observed in AC-treated mice. Furthermore, AC enhanced over 11% of SOD activities (*P* < 0.05,* F* = 5.16–12.24; Table 2) and 14% of GSH-Px activities (*P* < 0.05,* F* = 5.43–14.27; Table 2) in serum, liver, and skeletal muscle.

### 3.5. Effects of AC on the Activations of AMPK, Akt, and mTOR

**Figure 5:**
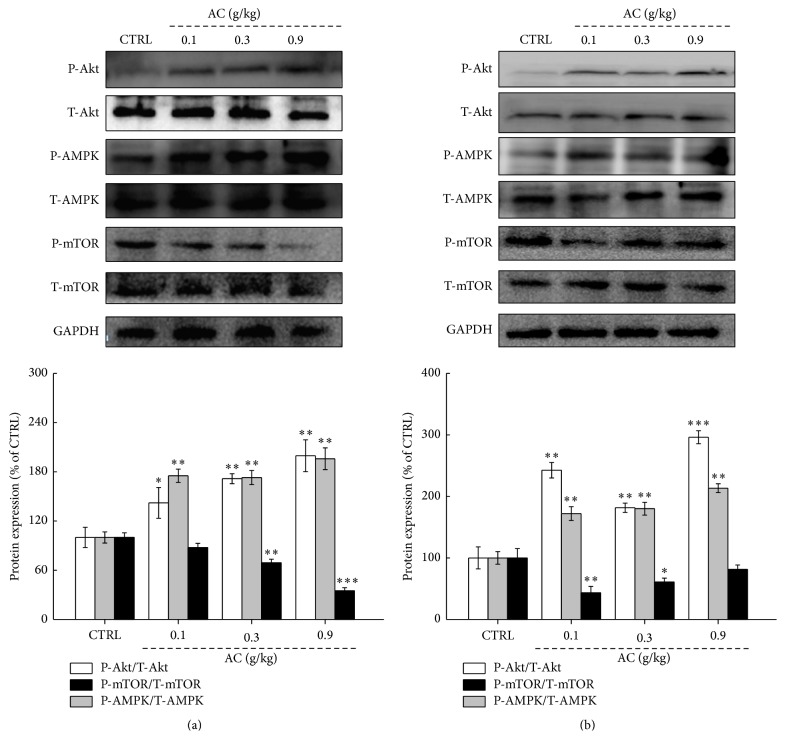
Three-week AC treatment upregulated the levels of phosphor-Akt and phosphor-AMPK and suppressed the expression of phosphor-mTOR in (a) livers and (b) skeletal muscle of mice with 60 min swimming. The data on quantified protein expression were normalized to the expressions of GAPDH. The data are expressed as means ± SEM (*n* = 6) and analyzed using a one-way ANOVA ^*∗*^*P* < 0.05, ^*∗∗*^*P* < 0.01, and ^*∗∗∗*^*P* < 0.001 in a comparison with the control mice.

To evaluate the potential mechanisms of AC on regulating energy metabolism and physical fatigue, the activations of AMPK, Akt, and mTOR in liver and skeletal muscle of mice after 60 min swimming were detected via western blot. In liver, compared to control mice, over 40% and 70% enhancement on P-Akt (142.1%–199.6% versus 100%; *P* < 0.05,* F* = 4.89–12.7) and P-AMPK expressions (173%–196% versus 100%; *P* < 0.01,* F* = 10.16–11.3) (Figure 5(a)) were noted in AC-treated mice. Moreover, AC reduced over 30% expressions on P-mTOR (69.2% and 35.1% versus 100%; *P* < 0.01,* F* = 11.49–26.3; Figure 5(a)). Detailed data can be found in Table  4s.

In skeletal muscle, the similar effects of AC on the activations of Akt, AMPK, and mTOR were noted as that in liver. Compared to control mice, AC enhanced over 80% and 70% P-Akt (181.6%–296.2% versus 100%; *P* < 0.01,* F* = 10.22–26.83) and P-AMPK expressions (172%–213.4% versus 100%; *P* < 0.01,* F* = 10.72–11.5) and reduced over 39% P-mTOR expressions (43.4% and 60.9% versus 100%; *P* < 0.05,* F* = 5.38–11.45) (Figure 5(b)). Detailed data can be found in Table  4s.

## 4. Discussion

The present study provided evidence that the oxidative stress may be the potential mechanism responsible for the antifatigue properties of AC. AC had no effect on the spontaneous activities of mice indicating its safety on central nervous system.* A. cinnamomea *has been used centuries in China and southeast Asia, in which its safety with few adverse effects has been emphasized. On the other hand, the crude drug nature of AC suggests multieffective components, displaying pharmacological activities via “systemically targets,” which may explain non-dose-dependent manner of AC observed in our experiments. The non-dose-dependent manner has been recognized as a common way of natural productions to show their pharmacological activities [32, 33].

Strenuous exercise is accompanied by the increased generation of free radical productions which contribute to oxidative stress [34]. Oxidative stress regulates the activity of the glycogen synthase kinase-3 and results in abnormalities of glucose and lipid metabolism, which is believed to be deeply involved in glycogen synthesis [35]. Glycogen is commonly subdivided into two types, hepatic glycogen that supports blood glucose concentration and muscle glycogen that provides muscle contraction for energy [36]. After strenuous exercise, hepatic glycogen metabolizes into glucose to support blood glucose consume; consequently, muscle glycogen metabolizes into lactic acid, which arrives in the liver and converts to hepatic glycogen or glucose used to replenish liver glycogen. Our present data display that a 60 min swimming exercise can cause hepatic glycogen consumption, and AC strongly enhanced the levels of glycogen in skeletal muscle and liver of mice with or without 60 min swimming.

In our study, 60 min swimming exercise caused oxidative stress by increasing ROS and MDA levels and decreasing the activity of GSH-Px. Importantly, AC strikingly decreased the ROS levels in mice with or without 60 min swimming. Moreover, an expected trend to decreased MDA levels and increased SOD and GSH-Px activities were noted in AC-treated mice. The overgeneration of ROS reacts with macromolecules including deoxyribonucleic acid (DNA), proteins, and the components of cellular membranes, which form lipid peroxidation products such as MDA [37]. MDA influences mitochondrial respiratory chain associated with the ATP synthase. MDA indirectly reflects the damage of membrane system and serves as an indicator of the degree of membrane liquid peroxidation [38]. Classically, two patterns of endogenous protective mechanisms have been described, enzymatic and nonenzymatic antioxidants, which help to reduce the injury of oxidative stress [39]. SOD and GSH-Px, the major components of enzymatic antioxidant defense systems, ameliorate exercise-induced fatigue by diminishing ROS [4]. SOD and GSH-Px combined together to scavenge free radicals, especially ROS, and inhibit lipid peroxidation by decreasing the production of MDA, thus to protect the cellular structures from destruction and further help to reducing fatigue [40]. Collectively, the regulating on accumulation and depletion of energy source related to antioxidative activities may be involved in AC-enhanced exercise tolerance.

AMPK plays a crucial role in the control of metabolism and energy synthesis and maintains the glucose homeostasis [41]. mTOR molecularly interacts with AMPK, constituting the operated switch of catabolism and anabolism [42]. Particularly, under the condition of adequate nutrition, the increased synthesis of ATP stimulates the activity of mTOR, which further promotes protein synthesis and lipogenesis [28]. Interestingly, AMPK positively replenishes cellular ATP supplies and suppresses the activation of mTOR to mediate ATP-consuming biosynthetic processes [43].* Gracilaria eucheumoides* [7] and* Paecilomyces hepiali *[24] extract improved the ability to fight fatigue in mice via modulating the activation of Akt, mTOR, and AMPK. Similarly, AC significantly inhibited the phosphorylation of mTOR by upregulating the activation of AMPK and Akt in liver and skeletal muscle, which may play central roles during its enhancing exercise tolerance.

There are still limitations in the present study. Firstly, we tried to find the different effects of AC on different genders, and our data found that AC showed similar antifatigue effects in male and female mice; however, more experiments need to be performed to confirm this conclusion. Secondly, we detected the changes of biochemical criterions in mice with or without swimming. Although we compared the differences between mice with and without exercise stimuli, we failed to get an accurate conclusion on the relationship between exercise and AC treatment, which will be further evaluated in our group.

In conclusion, AC, a natural nutritious product, strongly improves the exercise ability of mice correlated with inhibiting the depletion of glycogen stores, regulating oxidation-related enzymes, and modulating the activations of AMPK, Akt, and mTOR. Although our study is not comprehensive and requires further investigation, the evidences that we provide are to support the use of AC as a functional natural product against fatigue.

## Supplementary Material

Table 1s: Effects of AC on the excise ability of mice (Data of Figure 2).
Table 2s: AC regulated the activity of ALT and AST in the serum of mice without and with swimming (Data of Figure 3).
Table 3s: AC regulated the levels of glycogen in the tissue of mice without and with swimming (Data of Figure 4).
Table 4s: AC regulated the expression of Akt, AMPK and mTOR in the liver and skeletal muscle of mice (Data of Figure 5).

## Figures and Tables

**Table 1:** AC regulated the levels of ATP in the serum, liver, and skeletal muscle of mice with and without swimming.

		CTRL	AC (0.1 g/kg)	AC (0.3 g/kg)	AC (0.9 g/kg)
Serum (*μ*mol/gHb)	Without swimming	59.32 ± 6.02	69.36 ± 4.62	78.36 ± 4.81^*∗*^	75.35 ± 4.72^*∗*^
	With swimming	61.47 ± 5.86	86.7 ± 8.66^#∧^	89.33 ± 7.36^#^	97.11 ± 5.38^##∧∧^
Liver (*μ*mol/gprot)	Without swimming	67.35 ± 2.67	71.54 ± 4.79	80.64 ± 3.51^*∗∗*^	86.48 ± 4.4^*∗∗*^
	With swimming	86.01 ± 0.48^∧∧^	93.6 ± 10.52^∧∧^	92.41 ± 14.34^∧^	94.44 ± 18.89^∧^
Skeletal muscle (nmol/gprot)	Without swimming	18.06 ± 0.57	19.15 ± 0.53	20.55 ± 0.5^*∗*^	21.96 ± 0.64^*∗∗*^
	With swimming	19.1 ± 0.55	21.9 ± 0.33^#∧^	20.54 ± 0.33^#^	24.21 ± 0.39^##∧^

The data are expressed as means ± SEM (*n* = 12) and analyzed using a one-way ANOVA. ^*∗*^*P* < 0.05 and ^*∗∗*^*P* < 0.01 in a comparison with the control mice without swimming; ^#^*P* < 0.05 and ^##^*P* < 0.01 in a comparison with the control mice with 60 min swimming; ^∧^*P* < 0.05 and ^∧∧^*P*<0.01 in a comparison between the same agent treated mice with and without 60 min swimming.

**Table 2:** AC regulated the levels of ROS, MDA, GSH-Px, and SOD in the serum, liver, and skeletal muscle of mice with and without swimming.

	CTRL	AC (0.1 g/kg)	AC (0.3 g/kg)	AC (0.9 g/kg)
Serum				
Without swimming				
MDA (nmol/mL)	0.69 ± 0.07	0.47 ± 0.05^*∗*^	0.5 ± 0.05^*∗*^	0.58 ± 0.04
GSH-Px (U/mL)	39.93 ± 3.03	45.61 ± 1.97	47.07 ± 2.26^*∗*^	45 ± 2.87
SOD (U/mL)	33.26 ± 1.91	36.21 ± 1.08	41.27 ± 1.87^*∗∗*^	46.36 ± 0.86^*∗∗∗*^
With swimming				
MDA (nmol/mL)	0.90 ± 0.08^∧∧^	0.65 ± 0.08^#∧∧^	0.67 ± 0.05^#∧∧^	0.80 ± 0.05^∧∧^
GSH-Px (U/mL)	42.75 ± 2.14	51.64 ± 1.79^##∧^	48.79 ± 1.38^#^	45.43 ± 1.22
SOD (U/mL)	33.13 ± 1.58	35.80 ± 1.67	39.20 ± 1.85^#^	42.93 ± 1.07^##^

Liver				
Without swimming				
ROS (FI/mgprot)	222.90 ± 22.54	156.75 ± 26.84	128.11 ± 25.74^*∗*^	152.00 ± 19.32^*∗*^
MDA (nmol/mgprot)	0.78 ± 0.02	0.71 ± 0.03	0.68 ± 0.02^*∗*^	0.71 ± 0.01^*∗*^
GSH-Px (U/mgprot)	290.39 ± 17.32	323.8 ± 24.09	316.55 ± 13.23	322.83 ± 17.3
SOD (U/mgprot)	7.66 ± 0.41	8.4 ± 0.4	8.64 ± 0.43	8.65 ± 0.25^*∗*^
With swimming				
ROS (FI/mgprot)	290.67 ± 33.92^∧^	196.83 ± 36.54	183.88 ± 22.21^#∧^	187.29 ± 39.29^#^
MDA (nmol/mgprot)	0.83 ± 0.04	0.75 ± 0.03	0.74 ± 0.03	0.73 ± 0.03^#^
GSH-Px (U/mgprot)	282.31 ± 17.4	335.64 ± 21.31	333.72 ± 19.96	348.28 ± 18.36^#^
SOD (U/mgprot)	7.47 ± 0.37	8.21 ± 0.38	8.29 ± 0.44	8.60 ± 0.28^#^

Skeletal muscle				
Without swimming				
ROS (FI/mgprot)	106.89 ± 6.67	95.4 ± 6.18	83.1 ± 2.49^*∗∗*^	90.20 ± 0.71^*∗*^
MDA (nmol/mgprot)	9.63 ± 0.48	7.43 ± 0.53^*∗*^	7.56 ± 0.24^*∗*^	7.46 ± 0.21^*∗*^
GSH-Px (U/mgprot)	79.42 ± 1.75	85.19 ± 4.06	90.95 ± 1.95^*∗*^	91.57 ± 0.88^*∗∗*^
SOD (U/mgprot)	78.04 ± 1.22	80.11 ± 2.51	84.77 ± 1.43^*∗*^	83.36 ± 2.93
With swimming				
ROS (FI/mgprot)	111.78 ± 5.21	96.60 ± 5.18	95.88 ± 3.73^#^	97.22 ± 3.79^#∧^
MDA (nmol/mgprot)	9.13 ± 0.41	7.41 ± 0.45^#^	7.63 ± 0.33^#^	8.39 ± 0.33
GSH-Px (U/mgprot)	65.52 ± 3.19^∧^	76.61 ± 4.73^∧^	82.05 ± 4.8^#∧^	87.89 ± 6.17^#^
SOD (U/mgprot)	78.31 ± 1.59	93.35 ± 3.5^##∧^	83.56 ± 1.32	86.81 ± 1.55^#^

The data are expressed as means ± SEM (*n* = 12) and analyzed using a one-way ANOVA. ^*∗*^*P* < 0.05, ^*∗∗*^*P* < 0.01, and ^*∗∗∗*^*P* < 0.001 compared with control mice without swimming; ^#^*P* < 0.05 and ^##^*P* < 0.01 compared with control mice with 60 min swimming; ^∧^*P* < 0.05 and ^∧∧^*P*<0.01 in a comparison between the same agent treated mice with and without 60 min swimming.
