# Supplementary material for: Antifatigue Effects of Antrodia cinnamomea Cultured Mycelium via Modulation of Oxidative Stress Signaling in a Mouse Model
Source: Biomed Res Int. 2017 Mar 23;2017:9374026. doi: 10.1155/2017/9374026 (PMC5382311; doi:10.1155/2017/9374026)
Supplement: Supplementary file 1 — Table 1s: Effects of AC on the excise ability of mice (Data of Figure 2). Table 2s: AC regulated the activity of ALT and AST in the serum of mice without and with swimming (Data of Figure 3). Table 3s: AC regulated the levels of glycogen in the tissue of mice without and with swimming (Data of Figure 4). Table 4s: AC regulated the expression of Akt, AMPK and mTOR in the liver and skeletal muscle of mice (Data of Figure 5). [file 9374026.f1.pdf]

1. Materials and Methods

1.1 Animals experiments process

The experiments were approved by the Institution Animal Ethics Committee of Jilin University (Reference NO. 2015-003). 96 Kunming (KM) mice (8 weeks; 18-22 g; half male and female) were maintained under standard conditions (temperature 22°C±2°C, humidity 50%, 12 h light-dark cycle). Mice were randomly divided into 4 groups (n=24/group; F=0.014-1.37; half male and female) and orally treated with 10 mL/kg of normal saline (CTRL) and AC at doses of 0.1, 0.3, 0.9 g/kg daily for three weeks. The detailed experimental protocol and drug administration are shown in Fig.1.

1.1.1 Autonomic activities test. Thirty minutes after AC administration, mice were placed individually in the autonomic activities instrument (ZZ-6, Chengdu Taimeng Science Technology Co., Ltd., Chengdu, China). Locomotor activities and enabled vertical movements, including jumping, horizontal movements and walking and running, were counted.

1.1.2 Weight-loaded forced swimming Test (FST). Thirty minutes after AC administration, test was carried out with mice loaded with a lead block (10% of bodyweight) attached to their tails in water maintained at 25±2˚C. Mice loss coordinated movements and failure to swim to the surface within 10 sec were used to measure their exhaustion. The time of exhaustive swimming was recorded.

1.1.3 Rota-rod test (RRT). Before the formal test, mice were trained twice on rota-rod at 15 rpm for 60 s to adapt to the instrument (ZB-200, Chengdu Taimeng Science Technology Co., Ltd., Chengdu, China). And then, mice were placed inside a rota-rod spinning and allowed to run at speed of 20 rpm until they were exhausted and dropped from the rod. The total running time was recorded.

1.1.4 Exhaustive running test (ERT). Thirty minutes after AC administration, mice were trained twice on the runway at 20 rpm for 5 min to adapt to the treadmill (FT-200, Chengdu Taimeng Science Technology Co., Ltd., Chengdu, China). Exhaustion was determined by failuring to return to the runway within 15 sec and lossing dynamism of movements. The exhaustive running time was recorded.

1.2 Samples collection and biochemical indexes measurement

Thirty minutes after the final AC administration, half of mice (n=12/group; half male and female) were separately placed in the swimming pond (diameter 20 cm, depth 50 cm, and temperature 25±2°C) and swam for 60 min without loads. Another half of

mice (n=12/group; half male and female) were not received any treatment during 60 min. Blood, liver and skeletal muscle were collected and immediately placed in -80°C.

Levels of alanine aminotransferase (ALT) and aspartate aminotransferase (AST) in the serum, levels of glycogen in serum, liver and skeletal muscle were determined according to the protocol recommended by the commercial diagnostic kits purchased from Nanjing Jiancheng Institute of Biotechnology Co. Ltd. (Nanjing, China).

1.3 Western blot

Liver and skeletal muscle tissues were homogenized, lysed and measured total protein concentration by BCA protein assay kit (Merck Millipore, Germany). Samples containing 40 μg of total protein were loaded and separated by 12% SDS–PAGE (Bio-Rad, USA), transferred to nitrocellulose membrane (0.45 μm; Millipore, USA), and blocked for more than 4 h with 5% bovine serum albumin (BSA)/TBS buffer. Immunoblotting was detected using primary antibodies including phosphor (P)-Akt (07-1398), total (T)-mTOR (04-385), P-mTOR (09-213) (Merck Millipore, Darmstadt, Germany), T-Akt (ab131443), T-AMPK (ab133348) and P-AMPK (ab133348) (Abcam, Cambridge, USA) and glyceraldehyde-3-phosphate dehydrogenase (GAPDH) (sc-25778) (Santa Cruz Biotechnology, Santa Cruz, USA) via incubation overnight at 4°C followed by washing in TBST Buffer containing 5% BSA and 1% Tween-20. The primary antibody was detected with a HRP-conjugated goat anti-rabbit secondary antibody (sc-3836) (Santa Cruz Biotechnology, Santa Cruz, USA) and visualized by Gel Imaging System (UVP, California, USA). The bands density was quantified using Image J (National Institutes of Health, Bethesda, USA) via calculated the average optical density.

1.4 Statistical analysis

Data were expressed as mean ±S.E.M.. A one-way analysis of variance (ANOVA) was used to detect statistical significance followed by post-hoc multiple comparisons (Dunn's test) using SPSS 16.0 software (IBM corporation, Armonk, USA). P values less than 0.05 were considered significant.

2 Results

2.1 Effects of AC on mouse exercise capacity

Behaviors were measured to investigate the effect of AC on mouse exercise capacities. Compared to control mice, three-week AC treatment brought no significant differences in numbers of standing and activities indicating its safety on mice (P >

0.05, F = 0.03-1.47; Table.1s). FST, RRT and ERT are classic rodent models to evaluate the capacity of antifatigue. AC at doses of 0.3 and 0.9 g/kg strongly enhanced exercise capacity of mice in ERT and FST ($P < 0.05$, Table.1s). 0.9 g/kg of AC prolonged over 35% of exhaustive time than that of control mice in ERT (53.9 min vs. 39.8 min; $P < 0.01$, F = 10.23; Table.1s). In FST, 0.3 and 0.9g/kg of AC resulted in over 66% enhancement on swimming time than that of control mice (261.5 s and 252.8 s vs. 152.4 s; $P < 0.01$; F = 10.85 and 11.15; Table.1s). The retention time in rotating exercise was significantly increased by AC at 0.1 g/kg (6.4 min vs. 3.9 min; $P < 0.05$; F = 5.09; Table.1s).

2.2 Effects of AC on liver function

The levels of ALT and AST in the serum are considered as a biochemical marker for assessing liver function, which were examined to explore effect of AC on hepatic function. Compared to non-excise mice, 60-min swimming failed to influence the levels of ALT and AST in serum ($P > 0.05$, F = 0.18-1; Table.2s). In mice without swimming, AC at 0.3 g/kg strongly reduced the levels of ALT (5.89 IU/L vs. 10.31 IU/L; $P < 0.05$, F = 5.63) and AST (18.27 IU/L vs. 29.83 IU/L; $P < 0.05$, F = 4.43) (Table.2s). In mice with 60-min swimming, AC at 0.3 g/kg showed similar reductive effects on the serum levels of ALT (6.97 IU/L vs. 12.27 IU/L; $P < 0.05$, F = 6.5) and AST (18.77 IU/L vs. 28.74 IU/L; $P < 0.05$, F = 5.86) (Table.2s).

2.3 Effects of AC on levels of glycogen

Depletion of glycogen is the primary factor in fatigue and exhaustion during exercise. Compared to non-excise mice, low hepatic glycogen levels were observed in mice after 60-min swimming ($P < 0.05$, F = 4.85 and 5.3; Table.3s); however, no significant changes on muscle glycogen were noted in exercised mice ($P > 0.05$, F = 0.02-0.36; Table.3s). AC strongly enhanced the levels of hepatic glycogen and muscle glycogen in mice with and without swimming ($P < 0.05$, F = 4.39-6.11; Table.3s), but failed to influence the changes caused by excise stimuli. In mice with 60-min swimming, AC at 0.9 g/kg enhanced 15.5% of hepatic glycogen (11.2 mg/g vs. 9.7 mg/g; $P < 0.05$, F = 5.8), and 7.5% of muscle glycogen (2.33 mg/g vs. 2.21 mg/g; $P < 0.05$, F = 4.39) (Table.3s).

2.4 Effects of AC on the activations of AMPK, Akt and mTOR.

To evaluate the potential mechanisms of AC on regulating energy metabolism and physical fatigue, the activations of AMPK, Akt and mTOR in liver and skeletal muscle of mice after 60-min swimming were detected via western blot. In liver,

compared to control mice, over 40% and 70% enhancement on P-Akt (142.1%-199.6%vs. 100%; $P < 0.05$, $F = 4.89$-12.7) and P-AMPK expressions (173%-196% vs. 100%; $P < 0.01$, $F = 10.16$-11.3) (Table.4s) were noted in AC-treated mice. Moreover, AC reduced over 30% expressions on P-mTOR (69.2% and 35.1% vs. 100%; $P < 0.01$, $F = 11.49$-26.3).

In skeletal muscle, the similar effects of AC on the activations of Akt, AMPK and mTOR were noted as that of in liver. Compared to control mice, AC enhanced over 80% and 70% P-Akt (181.6%-296.2% vs. 100%; $P < 0.01$, $F = 10.22$-26.83) and P-AMPK expressions (172%-213.4% vs. 100%; $P < 0.01$, $F = 10.72$-11.5), and reduced over 39% P-mTOR expressions (43.4% and 60.9% vs. 100%; $P < 0.05$, $F = 5.38$-11.45) (Table.4s).

Table list

Table.1s Effects of AC on the excise ability of mice (Data of Fig. 2)

	CTRL	AC (0.1 g/kg)	AC (0.3 g/kg)	AC (0.9 g/kg)
Numbers of standing	50.33 ±3.83	50.27 ±3.79	49.74 ±3.18	50.35 ±4.06
Numbers of activities	12.63 ±1.16	12.91 ±1.36	12.10 ±1.48	12.95 ±1.31
Running time (min)	39.84 ±2.52	42.13 ±2.03	50.90 ±1.70*	53.93 ±1.19**
Swimming time (sec)	152.41 ±14.06	174.10 ±14.87	261.45 ±28.18**	252.77 ±22.02**
Rotating time (min)	3.93 ±0.74	6.35 ±0.77*	5.78 ±0.77	5.79 ±0.64

The data are expressed as means ± S.E.M. (n = 24) and analyzed using a one-way ANOVA. * $P < 0.05$ and ** $P < 0.01$ compared with control mice.

Table.2s AC regulated the activity of ALT and AST in the serum of mice without and with swimming (Data of Fig. 3)

		CTRL	AC (0.1 g/kg)	AC (0.3 g/kg)	AC (0.9 g/kg)
ALT (IU/L)	Without swimming	10.31±1.29	8.25±0.33	5.89±0.93*	7.88±0.78
	With swimming	12.27±1.28	8.85±0.93	6.97±1.18#	7.14±1.24
AST (IU/L)	Without swimming	29.83±2.37	22.54±2.17	18.27±2.34*	21.72±2.47
	With swimming	28.74±3.29	20.13±1.86	18.77±1.57#	17.65±1.72

The data are expressed as means ± S.E.M. (n = 12) and analyzed using a one-way ANOVA. * $P < 0.05$ in a comparison with the control mice without swimming; # $P < 0.05$ in a comparison with the control mice with 60-min swimming.

Table.3s AC regulated the levels of glycogen in the tissue of mice without and with swimming (Data of Fig. 4)

		CTRL	AC (0.1 g/kg)	AC (0.3 g/kg)	AC (0.9 g/kg)
Hepatic glycogen (mg/g)	Without swimming	10.61 ±0.29	12.37 ±0.54*	12.29 ±0.56*	12.47 ±0.62*
	With swimming	9.7 ±0.23	10.45 ±0.31^	10.33 ±0.44^	11.2 ±0.66#
Muscle glycogen (mg/g)	Without swimming	2.13 ±0.02	2.26 ±0.04*	2.29 ±0.03*	2.31 ±0.03*
	With swimming	2.21 ±0.02	2.29 ±0.05	2.24 ±0.02	2.33 ±0.02#

The data are expressed as means ± S.E.M. (n = 12) and analyzed using a one-way ANOVA. * $P < 0.05$ in a comparison with the control mice without swimming; # $P < 0.05$ in a comparison with the control mice with 60-min swimming; ^ $P < 0.05$ in a comparison between the same treated mice with and without 60-min swimming.

Table.4s AC regulated the expression of Akt, AMPK and mTOR in the liver and skeletal muscle of mice (Data of Fig. 5)

		CTRL	AC (0.1 g/kg)	AC (0.3 g/kg)	AC (0.9 g/kg)
Liver	P-Akt/T-Akt (% of CTRL)	100 ±12.31	142.1 ±18.79*	171.64 ±6.05**	199.55 ±19.37**
	P-AMPK/T-AMPK (% of CTRL)	100 ±6.84	175.23 ±8.07**	172.97 ±8.56**	195.98 ±13.25**
	P-mTOR/T-mTOR (% of CTRL)	100 ±5.56	87.80 ±5.03	69.17 ±4.19**	35.11 ±3.62***
Skeletal muscle	P-Akt/T-Akt (% of CTRL)	100 ±17.89	242.63 ±12.58**	181.62 ±7.57**	296.24 ±10.57***
	P-AMPK/T-AMPK (% of CTRL)	100 ±10.18	171.98 ±11.25**	180.01 ±10.36**	213.44 ±7.22**
	P-mTOR/T-mTOR (% of CTRL)	100 ±15.24	43.43 ±10.24**	60.92 ±6.24 *	81.3 ±7.21

The data are expressed as means ± S.E.M. (n = 6) and analyzed using a one-way ANOVA. * $P < 0.05$, ** $P < 0.01$ and *** $P < 0.001$ in a comparison with the control mice.
